# Case report of an adolescent girl with Kabuki syndrome and kyphoscoliosis, resistant at the conservative orthopedic treatment

**DOI:** 10.1186/1748-7161-7-S1-P4

**Published:** 2012-01-27

**Authors:** MA Taranu, M Colomer Giralt, V Calderón Padilla, V Pujol Blaya, L Quesada Morán, JM Cavanilles Walker, BM Núñez García, C Rodríguez Monje

**Affiliations:** 1 Hospital Germans Trias i Pujol, Badalona, Spain

## Background

The Kabuki syndrome (KS) is a rare genetic, hereditary, autosomic dominant, multiple anomaly syndrome, with an estimated incidence around 1-2/ 100 000 worldwide. Not all of the affected individuals have the same malformations.

Five major criteria delineate KS: postnatal short stature, skeletal anomalies, moderate mental retardation, dermatoglyphic anomalies, characteristic facial dysmorphism [1].

## Case report

We present the case of a 14 year girl diagnosed of the KS, refered to the Rehabilitation Service for kypho-scoliosis.

Searching in the literature, we didn’t find reported the association with kyphosis, but only scoliosis and unspecific vertebral malformations.

The first referal to our service was on May 2010, when the patient is 14 years 10 months old. The clinical examination: occipito-sacral axis centred, plumbline of C7, T6, L4, S1 - 3/0/ 6.5/0 cm, Adam’s Forward Bending test + (right rib cage elevated 2 cm), Hamstring tightness. Finger-floor distancy 25 cm.

The first Radiography: Hyperkyphosis 70°, Combined scoliosis T6-T12 12°, T11-L4 10°, Risser 2.

A first corse was prescribed - Maria Adelaida type, without correction neither of the scoliosis nor of the hyperkyphosis. A second corse Cheneau type was tried, without satisfactory results, and then a third corse, Michel Alegre modified type without improvement. The physiotherapy treatment was also prescribed. Presently, the case is to be evaluated by Raquis Unity Rehabilitation and Orthopedic Surgery.

## Conclusions

Is the Kyphosis in our patient a characteristic of the Kabuki Syndrome? Is it a consecuence of the Growth Hormone therapy our patient followed for 24 month for the short stature? Or is it a Scheuermann Disease accidentaly associated with the Kabuki syndrome?
